# Supplementary material for: Two Determinants of Dynamic Adaptive Learning for Magnitudes and Probabilities
Source: Open Mind (Camb). 2024 May 6;8:615–38. doi: 10.1162/opmi_a_00139 (PMC11093407; doi:10.1162/opmi_a_00139)
Supplement: Supplementary file 1 [file opmi-08-615-s001.pdf]

Supplementary Information for

Two determinants of dynamic adaptive learning for magnitudes and probabilities

Cedric Foucault and Florent Meyniel

cedric.foucault@gmail.com and florent.meyniel@cea.fr

Supplementary Text and Tables

Supplementary Text 1: Comparison between normative estimates and subjects' estimates.

Linear regression.

To see how subjects' estimates compared to normative estimates, we performed a linear regression between the two at the subject level, and then summarized the results at the group level. We collected two measures derived from the regression: the Pearson correlation coefficient and the slope of the linear regression. The results are reported in Tables S1 and S2 below.

The Pearson correlation coefficient results (Table S1) had already been reported in the main text. This coefficient measures the strength of the linear relationship between normative estimates and subjects' estimates. The fact that the coefficient is significantly greater than 0 shows that subjects' estimates covary with the optimal estimates, indicating that subjects perform the task adequately.

The slope of the linear regression indicates by how much the subject's estimate changes on average when the normative estimate changes by one unit. Many studies on human estimates, especially for probability judgments, have observed that the slope of the regression was less than 1 (Costello & Watts, 2014; Erev et al., 1994; Hilbert, 2012; Phillips & Edwards, 1966; Zhu et al., 2020). Consistent with these studies, we also observed in our study that the slope was less than 1, in both tasks (see Table S2 for descriptive and inferential statistics). In the literature, this phenomenon has been referred to as "conservatism bias" (Costello & Watts, 2014; Erev et al., 1994; Hilbert, 2012; Phillips & Edwards, 1966; Zhu et al., 2020), because a regression with a slope less than 1 predicts that, for a given level of normative estimate, the subject's estimate will be on average less close to the extremes (0 or 1, hence the 'conservatism' label), i.e. closer to 0.5, than the normative estimate. Here, we do not attach any particular mechanistic interpretation to the slope and treat it as a descriptive measure. For possible explanations of this phenomenon, see (Costello & Watts, 2014; Erev et al., 1994; Hilbert, 2012; Zhu et al., 2020).

Table S1. Pearson correlation coefficient between normative estimates and subjects' estimates.

Task	Mean	S.e.m.	Standard deviation	T-test against 0	
				t statistic	p value
Magnitude learning	0.96	0.01	0.09	106.20	2E-100
Probability learning	0.80	0.01	0.14	55.79	2E-74

Table S2. Slope of the linear regression between normative estimates and subjects' estimates.

Task	Mean	S.e.m.	Standard deviation	T-test against 0		T-test against 1	
				t statistic	p value	t statistic	p value
Magnitude learning	0.95	0.01	0.10	88.63	4E-93	4.79	6E-06
Probability learning	0.88	0.02	0.23	37.03	3E-58	4.96	3E-06

Decomposition of the mean squared error.

As presented in the main text, we performed a decomposition of the mean squared error between the subjects' estimates and the normative estimates to quantify the proportion of the error that was attributable to systematic biases in their estimates rather than to their variance (see Results).

We also conducted an additional analysis to investigate the bias: Since we observed a regression slope less than 1 consistent with a "conservatism bias" (Table S2), we investigated the extent to which such a conservatism bias could explain the subjects' bias. Specifically, we quantified the amount of bias explained by a linear regression model fitted to the subjects, which applies a linear transformation to the normative estimates, and models a conservatism bias when its slope is less than 1. We performed a linear regression between the normative estimates and the subjects' estimates averaged across the group, took the predictions of this regression as a model of the biased estimates, and then calculated the mean squared error obtained by replacing the normative estimates with the biased estimates. The proportion of the mean squared error that was reduced by using the biased estimates (i.e. the obtained reduction of the error in proportion to the original error) measures the amount of bias explained by the conservatism bias in subjects.

The full results of the decomposition (proportion of bias, variance, and of conservatism bias) are reported in Table S3 below.

Table S3. Decomposition of mean squared error between subjects' estimates and the normative estimates. MSE: mean squared error.

**Task**	**Proportion of MSE due to bias**	**Proportion of MSE due to variance**	**Standard error of the proportion of bias/variance**	**Proportion of MSE explained by the conservatism bias**	**Standard error of the proportion of conservatism bias**
Magnitude learning	23.48%	76.52%	1.12%	3.14%	0.63%
Probability learning	17.27%	82.73%	0.72%	3.23%	0.53%

Supplementary Text 2: Regression on subject's learning rate in the magnitude learning task performed as in a previous study.

For comparison with previous studies on magnitude learning, we additionally performed a regression analysis on the subject's learning rate as it was done in (McGuire et al., 2014), that is, without z-scoring regressors as we did in the regression reported in the main text, and replacing our prior uncertainty regressor by the RU*(1-CPP) regressor. We obtained regression weights similar to but slightly higher than those reported in (McGuire et al., 2014): The median and interquartile range of the regression weights in this analysis are 0.83 [0.56–0.92] and 0.51 [0.24–0.73] in our data for change-point probability and RU*(1-CPP) respectively (all two-tailed signed-rank $p<0.001$), vs. 0.53 [0.40–0.76] and 0.32 [0.11–0.44] in (McGuire et al., 2014).

Supplementary Text 3: Noisy delta-rule simulations

To examine the possibility that the learning rate adjustments observed in subjects could emerge from learning noise, we conducted simulations of a noisy delta rule model, with noise in the update similar to (Drugowitsch et al., 2016; Findling et al., 2019). The model is described by the following update equation:

$$v_t = v_{t-1} + \eta\ (x_t - v_{t-1}) + \varepsilon_t$$

where v_t is the model's estimate following observation x_t, η is the delta-rule parameter, and ε_t is the noise in the update, which is sampled from a zero-mean Gaussian distribution whose standard deviation corresponds to the noise level

We tested two variants for the noise level in the model: one (version a) where, as in (Drugowitsch et al., 2016), it is a constant parameter of the model, σ_ε, and another (version b) where, as in (Findling et al., 2019), it is scaled to the prediction error, with a scaling factor parameter ζ. Thus, the noise sampling is $\varepsilon_t \sim \mathcal{N}(0, \sigma_\varepsilon)$ in version (a) and $\varepsilon_t \sim \mathcal{N}(0, \zeta\ |x_t - v_{t-1}|)$ in version (b).

To compute the parameter values, we leveraged the following properties of the model (E denotes the expectation, SD the standard deviation):

$\eta = E[(v_t - v_{t-1}) / (x_t - v_{t-1})]$, since $E[\varepsilon_t / (x_t - v_{t-1})] = 0$.

$\sigma_\varepsilon = SD[(v_t - v_{t-1}) - \eta\ (x_t - v_{t-1})]$, for version (a).

$\zeta = SD[((v_t - v_{t-1}) - \eta\ (x_t - v_{t-1})) / |x_t - v_{t-1}|]$, for version (b).

By computing the means and standard deviations described above across time and sequences, we obtained, for each subject and task, the parameter values that best match the subject's estimates (Fig. S5B).

For the results obtained with this model regarding learning rate adjustments, see Fig. S5C.

Supplementary Text 4: Correlations across subjects between the magnitude learning task and the probability learning task.

We correlated the regression weights obtained in the magnitude learning task with those obtained in the probability learning task across subjects (the weights were obtained using the same regression as in Fig. 5). The weight of change-point probability was not significantly correlated between the two tasks (*r*=-0.01, p=0.92), and that of prior uncertainty was weakly though significantly correlated (*r*=0.26, p=0.012) (partial Pearson correlation controlling for the individual's average update frequency, two-tailed p values). This is indeed due to differences between the two tasks: within each task, when performing the same correlation analysis on two halves of the data (even and odd sessions), we obtained strong correlations (these were, in the magnitude and probability task respectively, *r*=0.64 and 0.95 for change-point-probability, *r*=0.38 and 0.74 for prior uncertainty, all p<0.001, two-tailed). For comparison, another behavioral measure, the average update frequency of the subject, was more strongly correlated across subjects between the two tasks: *r*=0.63, p<0.001 (Pearson correlation, two-tailed).

References

Costello, F., & Watts, P. (2014). Surprisingly rational: Probability theory plus noise explains biases in judgment. *Psychological Review*, *121*(3), Article 3. https://doi.org/10.1037/a0037010

Drugowitsch, J., Wyart, V., Devauchelle, A.-D., & Koechlin, E. (2016). Computational precision of mental inference as critical source of human choice suboptimality. *Neuron*, *92*(6), 1398–1411.

Erev, I., Wallsten, T. S., & Budescu, D. V. (1994). Simultaneous over-and underconfidence: The role of error in judgment processes. *Psychological Review*, *101*(3), 519.

Findling, C., Skvortsova, V., Dromnelle, R., Palminteri, S., & Wyart, V. (2019). Computational noise in reward-guided learning drives behavioral variability in volatile environments. *Nature Neuroscience*, 1–12. https://doi.org/10.1038/s41593-019-0518-9

Hilbert, M. (2012). Toward a synthesis of cognitive biases: How noisy information processing can bias human decision making. *Psychological Bulletin*, *138*(2), 211.

McGuire, J. T., Nassar, M. R., Gold, J. I., & Kable, J. W. (2014). Functionally dissociable influences on learning rate in a dynamic environment. *Neuron*, *84*(4), 870–881.

Nassar, M. R., Rumsey, K. M., Wilson, R. C., Parikh, K., Heasly, B., & Gold, J. I. (2012). Rational regulation of learning dynamics by pupil-linked arousal systems. *Nature Neuroscience*, *15*(7), 1040.

Nassar, M. R., Wilson, R. C., Heasly, B., & Gold, J. I. (2010). An approximately Bayesian delta-rule model explains the dynamics of belief updating in a changing environment. *Journal of Neuroscience*, *30*(37), 12366–12378.

Phillips, L. D., & Edwards, W. (1966). Conservatism in a simple probability inference task. *Journal of Experimental Psychology*, *72*(3), 346.

Prat-Carrabin, A., Wilson, R. C., Cohen, J. D., & da Silveira, R. A. (2021). Human Inference in Changing Environments With Temporal Structure. *Psychological Review*, *128*(5), 879–912. https://doi.org/10.1037/rev0000276

Vaghi, M. M., Luyckx, F., Sule, A., Fineberg, N. A., Robbins, T. W., & De Martino, B. (2017). Compulsivity reveals a novel dissociation between action and confidence. *Neuron*, *96*(2), 348–354.

Zhu, J.-Q., Sanborn, A. N., & Chater, N. (2020). The Bayesian sampler: Generic Bayesian inference causes incoherence in human probability judgments. *Psychological Review*, *127*(5), 719.

Supplementary Figures

Continued on next page

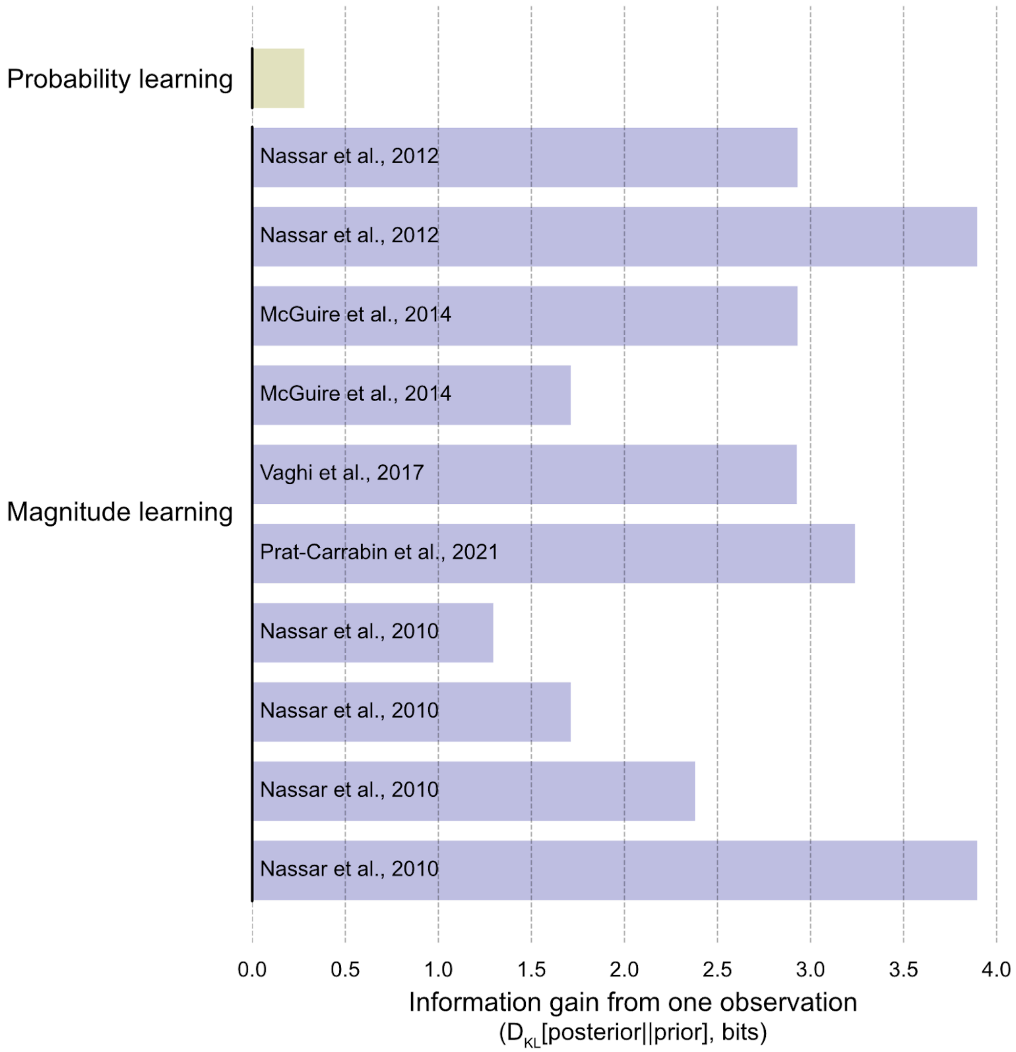

Fig. S1. Information gain provided by a single observation about the quantity to be learned in magnitude learning and probability learning. We computed the information gain as the KL divergence between the prior (uniform) distribution before having received any observation, and the posterior distribution about the underlying quantity after having received the observation, using the prior as reference distribution (i.e. D_{KL}[posterior||prior]), on average over the possible observations. The posterior is obtained from the prior and the likelihood function relating the observation to the underlying quantity using Bayes rule. In probability learning, the information gain is minimal. This is due to the binary nature of the observation. In magnitude learning, the information gain is larger because the observation is quantitative and typically fairly representative of the underlying magnitude. Although the latter depends on the experimenter's choice of standard deviation with which observations are generated, we computed the information gain for numerous experiments previously conducted and each condition of these experiments, and as shown above, in all cases it was substantially higher than that obtained in probability learning (McGuire et al., 2014; Nassar et al., 2010, 2012; Prat-Carrabin et al., 2021; Vaghi et al., 2017).

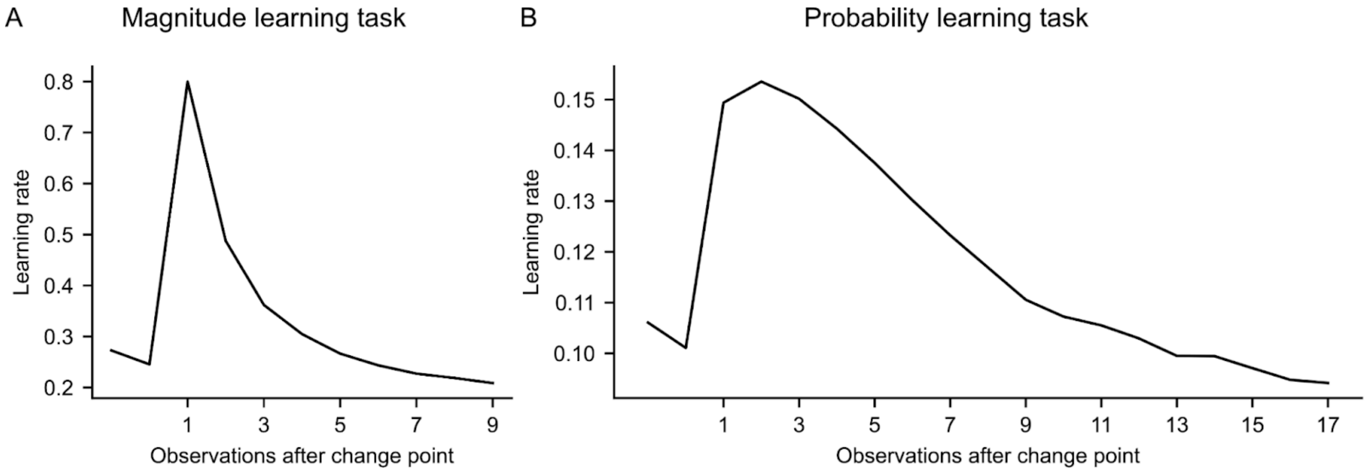

Fig. S2. Dynamics of the normative model's learning rate after a change point, in the magnitude (A) and probability (B) learning tasks. The plots were obtained as in Fig. 2, but rather than using the subjects' learning rate, we used the normative model's learning rate instead, which we obtained by running the normative model on the same sequences as the subject.

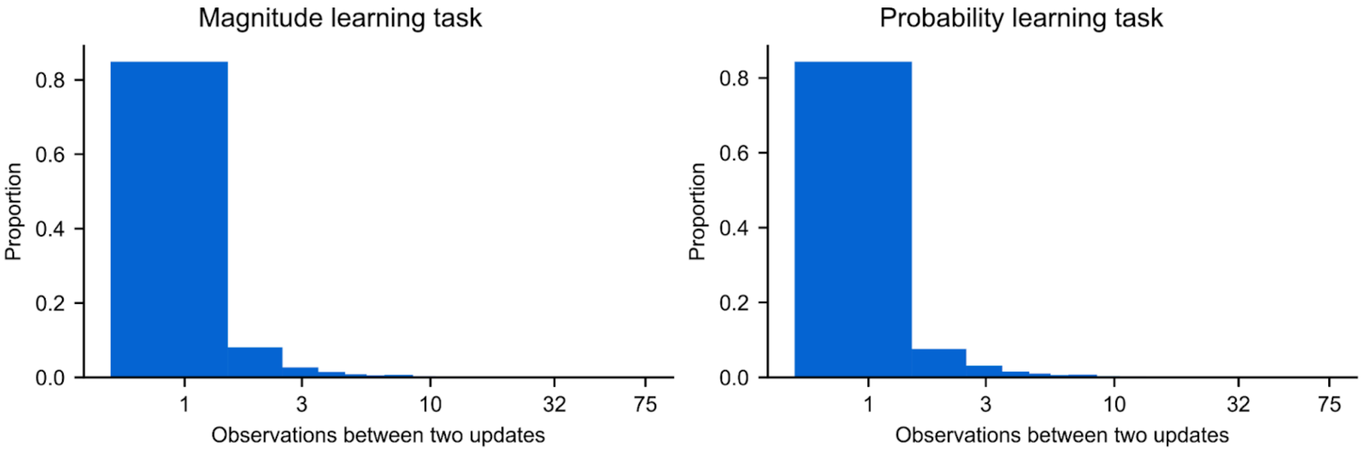

Fig. S3. Distribution of the number of observations elapsed between two report updates made by subjects. A log scale was used for the number of observations as in (Gallistel et al., 2014) for comparison (the equivalent distribution in Gallistel et al. is shown in their Fig. 11). In contrast to (Gallistel et al., 2014), in our study, updates were made on each observation most of the time (84% in the above distribution; mean of the distribution: 1.4 observation).

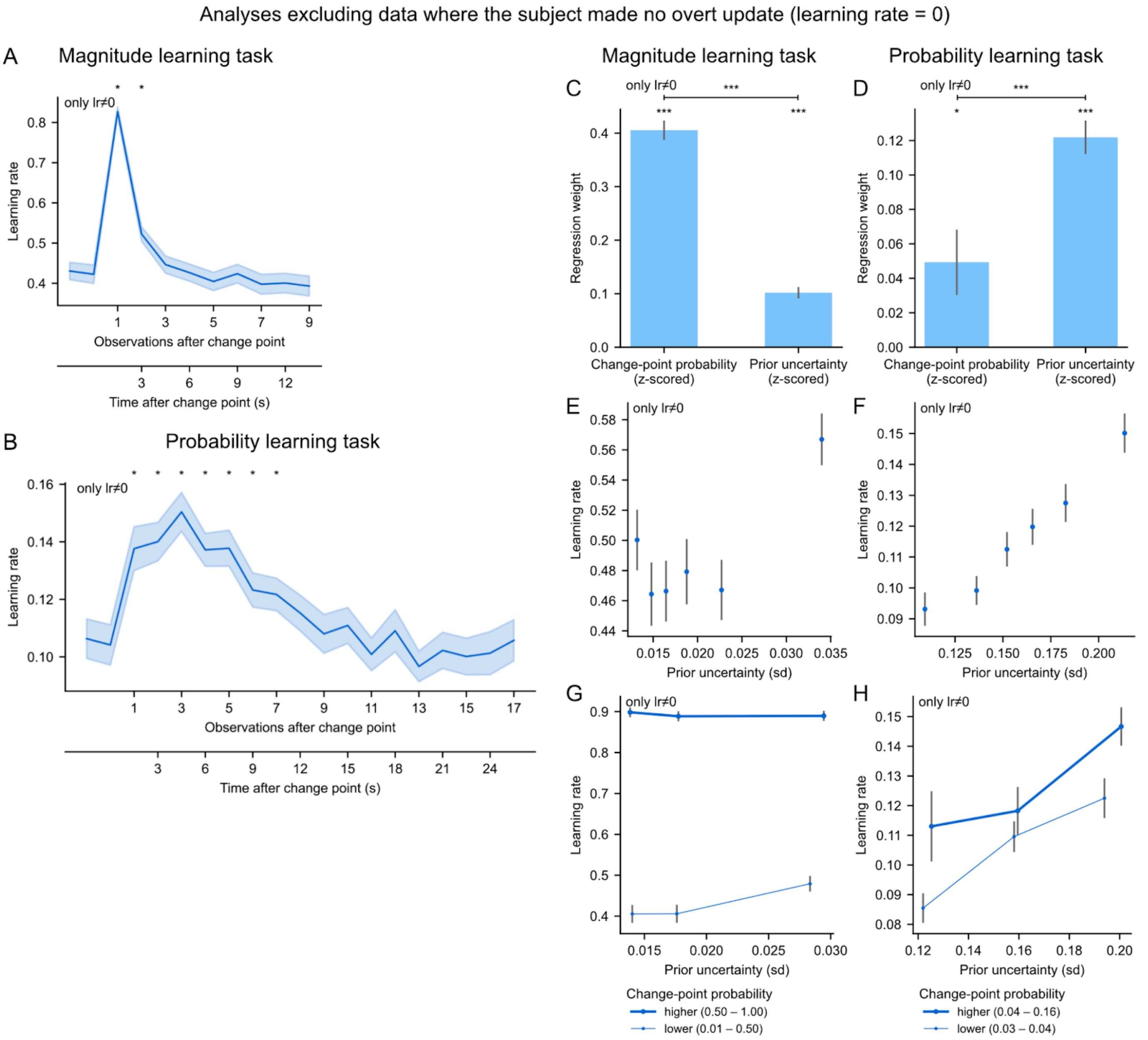

Fig. S4. The main results are similar and remain significant when excluding all data where the subject did not make an overt update. After excluding all data points where this was the case (i.e. learning rate = 0), we performed the same analyses as in previous figures and obtained the above plots: (A and B) Equivalent to Fig. 2 A and B; (C–H) Equivalent to Fig. 5 (A–F). Stars denote statistical significance as in the main figures (see legends of those figures for further details).

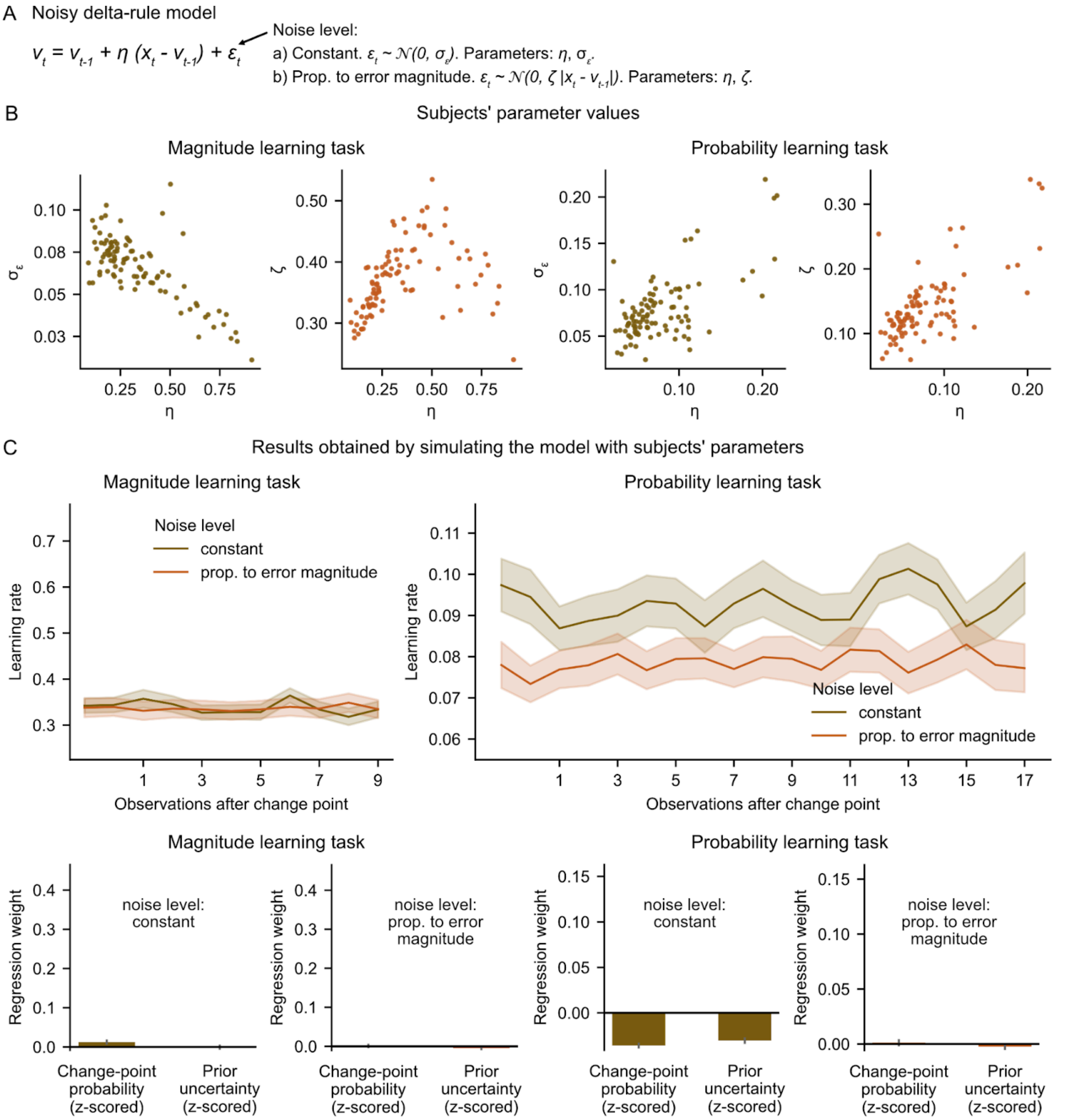

Fig. S5. The subjects' dynamic adjustments of the learning rate are not explained by learning noise. (A) Model of a delta-rule with learning noise. A noise sample is injected at each update of the model, otherwise governed by a delta-rule with parameter η. Two versions were tested for the noise level: (a) constant (parameter σ_ε), (b) scaled to the magnitude of the prediction error (scaling factor parameter ζ). (B) Values of the model parameters for each subject, for each version of the model and each task. Each dot represents one subject. (C) Results obtained by simulating the model with the subject's parameters on the subject's sequences and performing the same learning rate analyses as those reported in the main results for subjects. Top plots are the results for the analysis corresponding to Fig. 2, bottom plots to Fig. 5.

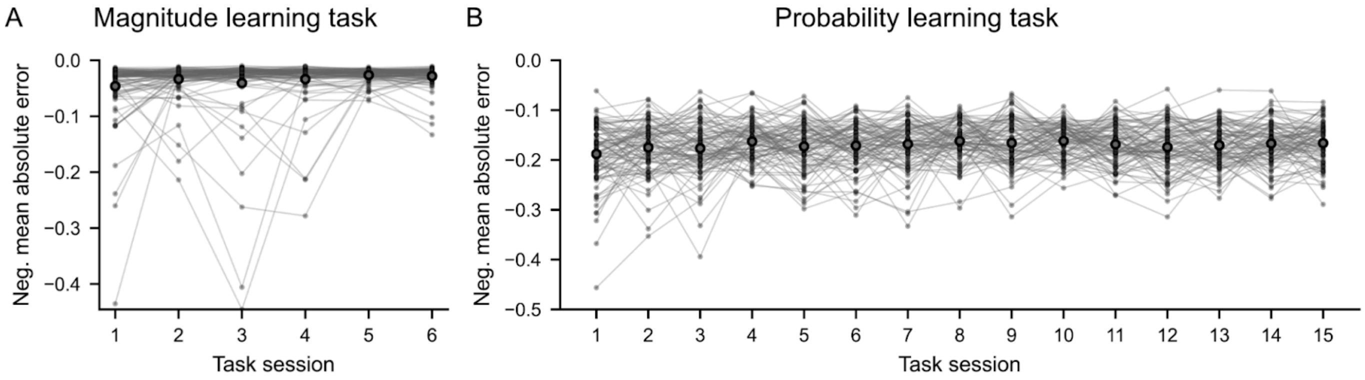

Fig. S6. Subjects' performance was stable over the course of the task. Performance is measured by the accuracy of the estimates, quantified by the mean absolute error between the subject's estimate and the true value of the hidden quantity (the negative of the error was used so that higher values correspond to higher performance). Thin dots and lines connecting them each denote one subject; large circles denote the mean across subjects.
